# Prevalence and Clinical Characteristics of Itch in Vitiligo and Its Clinical Significance

**DOI:** 10.1155/2017/5617838

**Published:** 2017-07-30

**Authors:** Vasanop Vachiramon, Woranit Onprasert, Sarawin Harnchoowong, Kumutnart Chanprapaph

**Affiliations:** Division of Dermatology, Faculty of Medicine Ramathibodi Hospital, Mahidol University, Bangkok, Thailand

## Abstract

**Objective:**

Vitiligo usually presented as asymptomatic depigmented macules and patches. Little is known regarding itch in vitiligo. This study aimed to evaluate the prevalence and characteristics of itch in vitiligo patients.

**Patients and Methods:**

A cross-sectional study was conducted on vitiligo patients. Itch character and intensity were determined through questionnaires. Evaluation was also made by dermatologists to define vitiligo subtype, body surface area, Koebner phenomenon (KP), and so on. Data were assessed by computer software. Results were considered statistically significant if *p* < 0.05.

**Results:**

Among 402 patients, itch on vitiliginous lesion presented in 20.2%. Prevalence of itch was most common in focal vitiligo (29.4%), followed by segmental vitiligo (20.3%) and nonsegmental vitiligo (19.6%), respectively. Tingling sensation was the most common itch-related symptom (82.7%). The median itch intensity is 5 by 10-point visual analog scale. Daily activity and sleep disturbance were observed in 60.5% and 39.5% of patients who experience itch. Itch occurred approximately 3 days prior to the development of lesions in 48.1% of patients. Thirty-two patients (78.1%) with both itch and KP type IIb had active disease.

**Conclusions:**

Itch in vitiligo is not uncommon. The presence of itch with KP type IIb may warrant the active vitiligo.

## 1. Introduction

Vitiligo is a common acquired hypopigmentary disorder, affecting up to 2% of the general population [1]. The pathogenesis of vitiligo is still not fully elucidated. However, it is postulated that vitiligo is a multifactorial, polygenic disorder, with a complex pathogenesis. Several theories on the etiology of vitiligo have emerged, for example, genetic hypothesis, autoimmune hypothesis, defects of melanocyte adhesion, neurogenic damage, and biochemical damage [2–7].

Classically vitiligo is manifested as well-defined, irregular-shaped, depigmented macules or patches. The lesions enlarge centrifugally over time, and the rate may be slow or rapid. Erythematous border is occasionally observed at the vitiliginous lesions, which is referred to as inflammatory vitiligo or vitiligo with raised inflammatory borders.

Vitiligo is usually considered as an asymptomatic dermatosis. However, itch was occasionally mentioned in some vitiligo patients. According to a study by Levai, vitiligo affected patients, with or without the presence of irritated skin lesions, can suffer from itch prior to the appearance of depigmented patches [8]. Furthermore, published data on the prevalence and characteristics of itch in vitiligo are very limited. The objective of this study was to determine the prevalence and factors influencing itch in patients with vitiligo.

## 2. Materials and Methods 

### 2.1. Study Design and Patient Eligibility

This questionnaire-based cross-sectional study was conducted from January 2016 to December 2016 at university-based hospital (Ramathibodi Hospital, Mahidol University, Bangkok, Thailand). Patients over 18 years of age affected with all types of vitiligo attending the dermatology outpatient department were enrolled in this study. They were excluded if they refused to participate in the study. Patients unable to read or understand the questionnaire were also excluded. The study was approved by institutional review board of Faculty of Medicine Ramathibodi Hospital, Mahidol University (protocol number 125813). The study protocol followed the guidelines of the 1964 Helsinki declaration. All patients had obtained the inform consent prior to enrollment.

### 2.2. Questionnaire Details

The questionnaire was divided into 2 parts. The first was self-reported information from patients, namely, demographic data (e.g., age, gender, weight, height, and income), family history of vitiligo, age of onset of vitiligo, initial location of the lesion, factors influencing the disease, Koebner phenomenon (KP) type 1, disease activity, and dermatology life quality index (DLQI). “True itch” refers to pruritus occurring before or after development of vitiliginous lesion but not related to treatment (e.g., topical medications, phototherapy). If it is present, patients then would be questioned about the different dimensions of itch including characteristic, aggravating and alleviating factors, the relationship of itch and the development of lesion, disease activity during the past 6 months, and sleep and activity disturbance. A 10-point visual analog scale (VAS) was used to grade itch intensity. Patients were also asked to compare the magnitude of itch to their past itch experience from a common itch-related condition, namely, insect bite. The VAS scale was categorized into the terms “mild itch” (VAS > 0 to <3.0), “moderate itch” (VAS ≥ 3.0 to <7.0), and “severe itch” (VAS ≥ 7.0 to 10).

The second part of the questionnaire was physician-reported information regarding physical examination and laboratory data of the participants, for example, Fitzpatrick skin type, other skin and systemic diseases, percentage of body surface area (BSA) involvement using the rule of nines as in burn assessment, type of vitiligo, leukotrichia, KP, anti-microsomal, anti-thyroglobulin, and anti-nuclear antibody (ANA), thyroid-stimulating hormone (TSH), free triiodothyronine (free T3), and free thyroxine (free T4).

KP was classified into 3 types based on the Vitiligo European Task Force group [9]. Type 1 KP is defined as the diagnosis of KP based on history (i.e., depigmentation after skin trauma). Type 2A KP is classified as patients having vitiligo localized to areas of repeated pressure or friction (e.g., elbows, knee, and knuckle) and type 2B KP is characterized by depigmented lesions clearly induced by trauma (i.e., artefactual lesions, punctiform, and crenate). Type 3 KP is defined as experimentally induced KP.

### 2.3. Assessment and Statistical Analyses

Statistical analyses were performed using computer software (Stata version 14, StataCorp., College Station, Texas) for Windows. Categorical variables were expressed as percentages. Continuous variables (e.g., age, BSA involvement, and VAS) were expressed in terms of mean ± standard deviation or median (range). Comparison of categorical variables was performed using the chi-squared test. The paired *t*-test or Wilcoxon signed-rank test was used to compare continuous data between two dependent samples. A *p* value of 0.05 or less was considered statistically significant.

## 3. Results

### 3.1. Patients' Demographic Data

A total of 402 patients were enrolled. There were 144 male and 258 female participants in the study. The mean age of patients was 45.43 (±16.19). The median duration of vitiligo was 7 years (0.8–59). Most patients had less than 20% of BSA involvement. Regarding type of vitiligo, nonsegmental vitiligo was the most common in this study (*N* = 321, 79.9%) followed by segmental vitiligo and focal vitiligo, 64 (15.9%) and 17 (4.2%), respectively. The demographic data and baseline characteristics of patients affected by vitiligo with and without itch are shown in Table 1. There were no significant differences in demographic characteristics between the two groups.

### 3.2. Prevalence of Itch and Itch Characteristics

Overall, itch was found in 81 patients with vitiligo (20.2%). Among vitiligo subtypes, itch was most commonly presented in focal vitiligo (29.4%), followed by segmental vitiligo (20.3%) and nonsegmental vitiligo (19.6%), respectively. The presence of itch significantly correlated with initial lesion located on the trunk (*p* < 0.001) (Table 1). Patients described the sensory qualities of itch as tingling (82.7%), crawling (18.5%), and burning (18.5%). Other itch sensations (e.g., pinching, electric discharge, and tickling) were rarely reported (<3%). Approximately 21% of patients who suffered from itch reported having at least two sensory qualities (Table 2). Among them, women were reported to have crawling sensation more frequently than men (7.4% in men versus 24.1% in women, *p* = 0.069) (Table 3).

### 3.3. Severity of Itch

The median intensity of itch on 10-point VAS was 5 (1–10), a magnitude equivalent to pruritus caused by insect bite reaction through the patients' experiences. Severe itch was reported in 23 patients (28.4%). Itch of moderate and mild intensity was documented in 35 (43.2%) and 23 (28.4%) of patients, respectively (Table 2). Itch was described to be more intense in females when compared to the male counterpart (33.3% versus 18.6%, *p* = 0.291) (Table 3).

**Figure 1:**
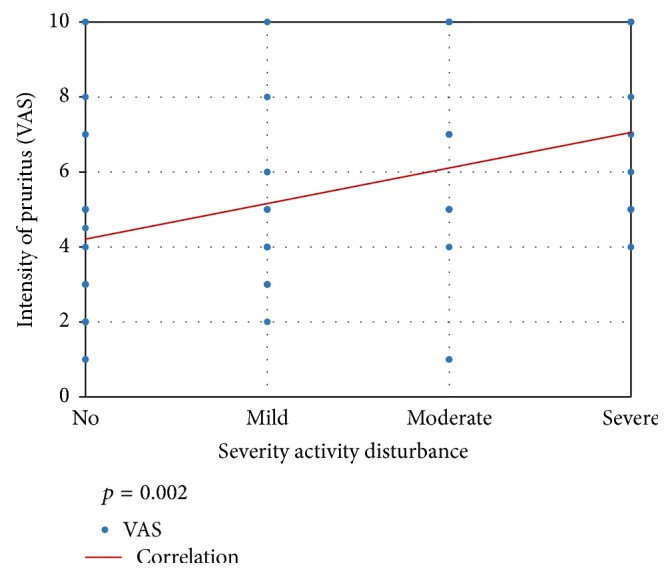
Correlation of intensity of pruritus and degree of activity disturbance.

As for the burden of itch among affected patients, the median DLQI score in the itch group was significantly higher than those without it (7 versus 5, *p* < 0.001) (Table 1). Daily activity disturbance was found in 49 patients (60.5%) and sleep disturbance was found in 32 patients (39.5%). Thirty patients (37%) reported moderate to severe activity disturbance. This was significantly more among female patients (*p* = 0.033) (Table 3). The magnitude of activity disturbance correlated significantly with itch intensity (*p* = 0.002) (Figure 1).

### 3.4. Relationship of Itch and the Development of Lesions

Itch preceded the onset of vitiliginous lesions in 39 patients (48.1%) with the median of 3 days (1–60) prior to the development of the lesion. Thirty-seven patients (45.7%) reported that itch occurred after the appearance of vitiliginous lesion, with the median of 1 day after the onset (0.5–30) (Table 2). In 5 out of 81 patients who had itch (6.2%), the relationship between onset of itch and the lesion development could not be determined.

### 3.5. Aggravating Factors and Alleviating Factors

Common aggravating factors of itch were skin dryness (58.0%), hot environment (49.4%), and sunlight (24.7%). Topical corticosteroids (55.6%) were the most common itch-alleviating factor followed by shower (38.2%). Oral antihistamine improved itch in some patients (9.9%). Cold environment was found to both aggravate and alleviate itch in 16.1% and 25.9% of patients, respectively. Factors that aggravated and relieved itch were shown in Table 2.

### 3.6. Itch and the Disease Activity

**Figure 2:**
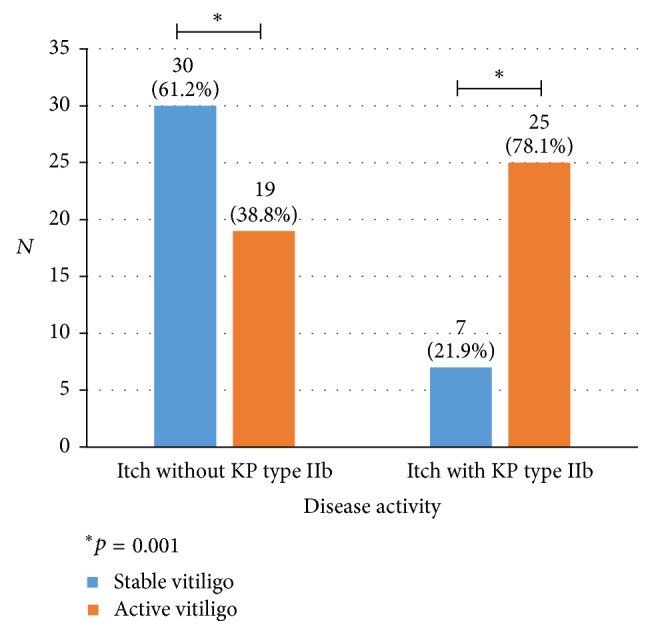
Disease activity among patients with itch and Koebner phenomenon (KP) type IIb.

In this study, KP was found in 56% of patients. The presence of KP types I and II tends to be more common in vitiligo patients with itch compared to those without. This is especially true for KP type IIb (*p* = 0.056) (Table 4). Among 32 patients with itch and KP type IIb, 78.1% had active disease (i.e., lesion progression and/or newly developed lesion), while 21.9% had stable disease within the past 6 months (*p* = 0.001) (Figure 2).

## 4. Discussion

Itch is a common symptom of many dermatological diseases. It may originate in the skin or nervous system. Clinically, itch can be classified into itch associated with skin disorders (e.g., xerosis, atopic dermatitis, urticaria, psoriasis, and arthropod bite), itch associated with systemic diseases (e.g., chronic liver disease, end stage renal failure), neuropathic itch (e.g., notalgia paresthetica, postherpes zoster neuropathy), and psychogenic itch. However, itch associated with vitiligo is rarely mentioned in the literature.

In this study, the prevalence of itch in vitiligo patients is up to 29.4% depending on vitiligo subtype. The prevalence of itch in this study is higher than a previous report. According to a study by Levai, 44% of vitiligo patients had a history of prior skin disease in an area of depigmentation, 15% had depigmentation in areas of constant pressure or irritation, and 10% complained of itching without prior history of skin disease or trauma [8]. The higher prevalence of itch in our study could be due to the differences in the study population. Itch in several patients with depigmented lesions described by Levai, in fact, could possibly be from chemical leukoderma and postinflammatory depigmentation and, thereby, may not represent the exact prevalence of itch in true vitiligo patients.

Itch can occur as a consequence of treatment, for example, topical mediation and phototherapy. We have excluded this group from the study. Moreover, certain systemic disease associated with vitiligo could potentiate itch such as hyperthyroidism, hypothyroidism, diabetes mellitus, allergic rhinitis, or asthma. According to our study, there were no significant differences regarding the prevalence of these conditions among patients with and without itch. Therefore, we postulate that itch in vitiliginous lesion is a manifestation of vitiligo itself.

The pathogenesis of itch in vitiligo remains unclear. However, the possible concept of itch in many dermatological diseases is focused on neurogenic inflammation and mediators such as neuropeptides released from dermal nerve endings induced by various stimuli. There are many evidences to support the hypothesis of inflammation in vitiliginous lesion. According to recent studies, cell-mediated immunity in vitiligo was demonstrated by the presence of CD8+ T cells in suction blistering skin, perilesional skin, and the blood of vitiligo patients. Moreover, vitiliginous lesion demonstrated lower CD4+ to CD8+ lymphocytes ratio compared to nonlesional skin [10–13]. Other parameters such as cytokines and regulatory T cells may also play major roles in vitiligo pathogenesis [14]. An increase in tumor necrosis factor alpha (TNF-*α*), interferon-gamma (IFN-*γ*), and interleukin- (IL-) 1 compared to healthy skin suggests that vitiligo is mediated by cytotoxic T cells and T helper cell-1 (Th1) response [15–18]. In segmental vitiligo, dysfunction of the sympathetic nervous system can restrain melanin production and lead to depigmentation [19]. All these findings may illustrate neurogenic inflammatory mechanism of itch in vitiligo. However, it is unclear whether primary immune response targets normal melanocytes or if immune activation is triggered by damaged melanocytes through exogenous or endogenous insult. Further studies are needed to explain the complex mechanisms of itch in vitiligo.

There are several methods to assess the intensity of itch. In this study, the ratings were based on patients' 10-point VAS self-evaluation scale. According to our data, the median intensity of itch in was 5 (median VAS = 5). Our patients rated this equivalent to the severity of itch caused by arthropod bite. However, the comparison itch intensity in different types of dermatoses is very difficult and no direct assessment has been performed. Based on previous studies, using a similar VAS score, the intensity of itch in atopic dermatitis, psoriasis, and lichen planus was 4.8, 6.3, and 6.9, respectively [20–22]. In this study, 71.6% of patients with itch reported moderate or severe intensity. Approximately 60% of vitiligo patients with itch had daily activity disturbance and 39.5% had sleep disturbance. This may result in psychological distress and potentiate the severity of several dermatoses including vitiligo. Therefore, we emphasized the importance of itch in vitiligo which remains underrecognized.

Among the clinical variants of vitiligo, pruritus was more common in focal vitiligo and significantly correlated with initial truncal distribution. Our finding supports previous evidence on the variations in area-specific innervation density and pruritic mediator release in different anatomical sites of the body [23, 24]. In addition, the nerves which supply sensation to the upper trunk emerge from the 2nd to 6th thoracic segments of spinal cord. They run a long course up through the thick muscles of the back and make a right-angled turn before reaching the skin. These nerves appear to be vulnerable to compression or traction and lead to the symptom of itch [25]. Although our data indicates that the presence of initial lesion on trunk is highly associated with itch (*p* = 0.001), the numbers are too small to make a convincing support that the location is the key issue for itch. When compared to other itchy dermatosis, pruritus in lichen planus tends to be more prominent on the extremities, while psoriatic patients experience itch on both the trunk and lower extremities [22, 26].

Regarding factor influencing itch, skin dryness was identified as an aggravating factor of pruritus by 58% of the patients. However, the presence of skin dryness did not correlate with the degree of itch which is consistent with other pruritic dermatoses [27–29]. Several factors such as alteration stratum corneum surface lipid, water metabolism, pH, and cytokine levels may contribute to the sensation of itch [30, 31].

Hot environment and sunlight were identified as an important aggravating factor of itch in our vitiligo patients, possibly from high temperatures in our tropical climate. It was suggested that heat can increase itch sensation by its direct effect on dermal nerve endings or by indirect effect on neuroautonomic mechanism via sweating, as both itch and sweating are mediated by C nerve fibers [32, 33]. According to recent data, heat stimulated itch through the activation of transient receptor potential cation channel subfamily vanilloid type 1 (TRPV1), the calcitonin gene-related peptide, the vesicular glutamate transporter 2, and accumulation of artemin [34]. Further investigations are needed to validate whether these receptors are involved in the pathogenesis of itch in vitiligo.

The most common antipruritic treatment used was topical steroids. Due to the evidence of inflammatory cell infiltration and certain cytokines in vitiliginous lesion, anti-inflammatory therapies may result in cessation of itch. Moisturizers in topical medication may have a contributing benefit in reducing the itch. In addition, cold environment and shower were found to alleviate itch in vitiligo. These observations support a role for the cold-sensitive transient receptor potential melastatin-8 (TRPM8) ion channel, the major receptor for sensing cold environmental, in the modulation of pruritus [35]. In contrast to other pruritic dermatoses, the role of antihistamines in relieving itch is questionable in vitiligo. Only a few of our patients responded to oral antihistamines.

According to a study by Van Geel et al., higher disease activity was found in KP1-positive and KP2B-positive vitiligo patients [9]. In our study, there is a tendency towards itch being more prevalent in patients presenting with KP type 2B (Table 4). Therefore, it could be implied that the presence of itch may warrant active stage of vitiligo especially with the presence of KP type IIb. A possible explanation to this phenomenon is that the inflammatory process of active vitiligo promotes pruritogenic cytokines and induces itch.

There are some limitations in our study. Firstly, recall bias associated with self-responded questionnaire may influence the results. Secondly, as patients were gathered from university-based hospital, there is a possibility of subject selection election bias. Thirdly, our study involved patient subjected evaluation; an objective assessment was not performed. Finally, further studies regarding the neurophysiology of itch including mechanism of itch in vitiligo are necessary.

In conclusion, this is the first step towards enhancing comprehensive knowledge regarding itch in vitiligo. As dermatologists, it is prudent to acknowledge that pruritus is an important aspect of vitiligo. Prompt detection to provide early treatment is mandatory in patients with active vitiligo.

## Figures and Tables

**Table 1:** Demographic data and clinical characteristics of vitiligo patients with and without itch.

Characteristics	Itch	Nonitch	*p*
Age, mean ± SD (years)	46.5 ± 16.0	45.2 ± 16.2	0.519

Gender, *n* (%)			0.601
(i) Male	27 (33.3)	117 (36.4)	
(ii) Female	54 (66.7)	204 (63.6)	

Onset (years), median (range)	37 (0–72)	36 (1–75)	0.438

Duration of vitiligo (years), median (range)	6 (0–59)	7 (0.83–51)	0.456

Family history of vitiligo, *n* (%)	17 (21.0)	73 (22.7)	0.735

Family history of thyroid and autoimmune disease, *n* (%)	10 (12.4)	40 (12.5)	0.978

Leukotrichia, *n* (%)	38 (46.9)	151 (47.0)	0.984

Body surface area involvement (%), median (range)	3 (0.2–50)	2 (0.1–90)	0.458

Fitzpatrick skin types, *n* (%)			0.521
(i) Type III	13 (16.0)	57 (17.8)	
(ii) Type IV	57 (70.4)	234 (72.9)	
(iii) Type V	11 (13.6)	30 (9.3)	

Type of vitiligo, *n* (%)			0.618
(i) Nonsegmental	63 (77.8)	258 (80.4)	
(ii) Segmental	13 (16.0)	51 (15.9)	
(iii) Focal	5 (6.2)	12 (3.7)	

Initial location, *n* (%)			
(i) Head and neck	37 (45.7)	143 (44.6)	0.855
(ii) Trunk	13 (16.1)	17 (5.3)	0.001
(iii) Arms	4 (4.9)	38 (11.8)	0.070
(iv) Hands	17 (21.0)	103 (32.1)	0.051
(v) Legs	9 (11.1)	23 (7.2)	0.241
(vi) Feet	5 (6.2)	12 (3.7)	0.354

Associated diseases, *n* (%)	28 (34.6)	110 (34.3)	0.959
(i) Hyperthyroidism	1 (2.6)	15 (9.6)	0.560
(ii) Hypothyroidism	1 (2.6)	11 (6.7)	0.560
(iii) Diabetes mellitus	3 (3.7)	14 (4.4)	1.000
(iv) Allergic rhinitis/asthma	6 (7.4)	22 (6.9)	0.861
(v) Alopecia areata	1 (1.2)	9 (2.8)	0.368
(vi) Halo nevi	3 (3.7)	19 (5.9)	0.319

Positive results for anti-microsomal antibody	16 (25.4)	75 (30.0)	0.472
Positive results for anti-thyroglobulin antibody	12 (19.5)	49 (19.8)	0.888
Positive results for ANA	30 (45.5)	117 (45.2)	0.967

TSH level			0.216
(i) Low	2 (3.23)	16 (7.31)	
(ii) Normal	60 (96.77)	195 (89.04)	
(iii) High	0 (0)	8 (3.65)	

DLQI, median (range)	7 (0–26)	5 (0–26)	0.001

**Table 2:** Characteristics of itch in vitiliginous lesions.

Characteristics	*n* (%)
Prevalence of itch	81 (20.2), 95% CI (16.3–24.4)
Intensity of itch in vitiligo (VAS), median (range)	5 (1–10)
Intensity of itch in insect bite (VAS), median (range)	5 (0–10)
Frequency of itch	
(i) Always	4 (4.9)
(ii) Often	77 (95.1)
Aggravating factors	
(i) Hot environment	40 (49.4)
(ii) Dry skin	47 (58.0)
(iii) Sunlight	20 (24.7)
(iv) Cold environment	13 (16.1)
(v) Seafood	7 (8.6)
Alleviating factors	
(i) Topical corticosteroids	45 (55.6)
(ii) Shower	31 (38.2)
(iii) Cold environment	21 (25.9)
(iv) Oral antihistamines	8 (9.9)
(v) Phototherapy	5 (6.2)
Relationship of itch and onset of lesion(s)	
(i) Itch before onset	39 (48.1)
Day, median (range)	3 (1–60)
(ii) Itch after onset	37 (45.7)
Day, median (range)	1 (0.5–30)
(iii) Uncertain	5 (6.2)
Itch sensory qualities	
(i) Tingling	51 (63.0)
(ii) Crawling	5 (6.2)
(iii) Burning	7 (8.7)
(iv) Pinching	1 (1.2)
(v) Tingling + crawling	5 (6.2)
(vi) Tingling + burning	4 (4.9)
(vii) Tingling + electric discharge	1 (1.2)
(viii) Tingling + tickling	2 (2.5)
(ix) Tingling + crawling + burning	4 (4.9)
(x) Crawling + electric discharge	1 (1.2)
Severity of itch	
(i) Mild itch (VAS > 0 to <3.0)	23 (28.4)
(ii) Moderate itch (VAS ≥ 3.0 to <7.0)	35 (43.2)
(iii) Severe itch (VAS ≥ 7.0 to 10).	23 (28.4)

**Table 3:** Intensity, sensation of pruritus, and activity and sleep disturbance between males and females.

Characteristics	Male, *n* (%)	Female, *n* (%)	*p*
Intensity of pruritus in vitiliginous lesion by VAS			0.291
(i) Mild (1–3)	10 (37.0)	13 (24.1)	
(ii) Moderate (4–6)	12 (44.4)	23 (42.6)	
(iii) Severe (7–10)	5 (18.6)	18 (33.3)	
Itch sensory qualities			
(i) Tingling	24 (88.9)	43 (79.6)	0.365
(ii) Crawling	2 (7.4)	13 (24.1)	0.069
(iii) Burning	6 (22.2)	9 (16.7)	0.544
(iv) Pinching	0	1 (1.9)	1.000
(v) Electric discharge	1 (3.7)	1 (1.9)	1.000
(vi) Tickling	1 (3.7)	1 (1.9)	1.000
Activity disturbance			0.033
(i) No	15 (55.6)	17 (31.4)	
(ii) Mild	8 (29.6)	11 (20.4)	
(iii) Moderate	2 (7.4)	15 (27.8)	
(iv) Severe	2 (7.4)	11 (20.4)	
Sleep disturbance			0.152
(i) No	21 (77.8)	28 (51.9)	
(ii) Mild	4 (14.8)	14 (25.9)	
(iii) Moderate	1 (3.7)	6 (11.1)	
(iv) Severe	1 (3.7)	6 (11.1)	

**Table 4:** Koebner phenomenon (KP) between itch and nonitch groups.

Koebner phenomenon	Itch (*n* = 81)	Nonitch (*n* = 321)	*p*
KP, *n* (%)	50 (67.9)	175 (54.52)	0.243
KP type I	35 (43.2)	110 (34.3)	0.134
(i) Cutting/scratching	28 (34.6)	86 (26.8)	0.165
(ii) Rubbing	35 (8.7)	24 (7.5)	0.082
(iii) Chemical/heat/cold	5 (6.2)	16 (5.0)	0.588
(iv) Pressure	5 (6.2)	8 (2.5)	0.094
(v) Previous skin disease	4 (4.9)	11 (3.4)	0.521
KP type II	43 (53.1)	141 (43.9)	0.139
(i) KP type IIa	19 (23.46)	76 (23.8)	0.967
(ii) KP type IIb	32 (39.5)	92 (28.9)	0.059
KP type III	1 (1.23)	4 (1.25)	1.000
